# Fatal Opioid Overdoses by Historical and Contemporary Neighborhood-Level Structural Racism

**DOI:** 10.1001/jamahealthforum.2025.3986

**Published:** 2025-11-07

**Authors:** Mudia Uzzi, Jordyn R. Ricard, Imani Belton, Sabriya Linton, Lea Marineau, Renee M. Johnson, Carl Latkin, Elizabeth Nesoff

**Affiliations:** 1 Department of Health Policy and Management, Johns Hopkins Bloomberg School of Public Health, Baltimore, Maryland; 2 Department of Mental Health, Johns Hopkins Bloomberg School of Public Health, Baltimore, Maryland; 3 Department of Psychology, Yale University, New Haven, Connecticut; 4 Department of Behavioral, Social and Health Education Sciences, Emory University, Rollins School of Public Health, Atlanta, Georgia; 5 Department of Psychiatry and Behavioral Sciences, Johns Hopkins University School of Medicine, Baltimore, Maryland; 6 Department of Health, Behavior and Society, Johns Hopkins Bloomberg School of Public Health, Baltimore, Maryland; 7 University of Pennsylvania Perelman School of Medicine, Philadelphia

## Abstract

**Importance:**

Black, Indigenous, and Latino communities are disproportionately affected by the US overdose epidemic. Structural inequalities, encompassing social, economic, and infrastructural dimensions, have been increasingly theorized as fundamental drivers of these disparities.

**Objective:**

To investigate whether there is an association between neighborhood-level structural racism and opioid-involved overdose deaths in an urban area.

**Design, Setting, and Participants:**

This ecological serial cross-sectional study of 796 census tracts (2017-2019) and 792 census tracts (2020-2022) in Chicago, Illinois, used a geospatial and intersectional analytic approach. A quasi-Poisson spatial regression was conducted to examine associations between neighborhood-level structural racism and census tract–level opioid-involved overdose deaths before the COVID-19 pandemic (2017-2019) and during the COVID-19 pandemic (2020-2022). Eigenvector spatial filtering was used to control for residual spatial autocorrelation. Population density was also accounted for in the regression model. Two structural racism indicators (historical redlining and contemporary racialized economic segregation) were combined to develop an index that captures 4 distinct neighborhood intersectional groups of racism over an 80-year period. Average marginal effect calculations were also performed to support the interpretability of the findings. Data were analyzed from February 19, 2024, to July 3, 2025.

**Exposure:**

A combined measure of 2 structural racism indicators (historical redlining and contemporary racialized economic segregation).

**Main Outcomes and Measures:**

Overdose deaths were aggregated to census tracts; the main outcome measure was the number of overdose deaths at the census tract-level.

**Results:**

The total sample sizes were 796 census tracts before the COVID-19 pandemic (2017-2019) and 792 census tracts during the COVID-19 pandemic (2020-2022). As defined by the study’s combined measure of structural racism, census tracts with high levels of racism in the past and/or present showed statistically significantly higher number of fatal overdoses compared with tracts with low levels of racism both in the past and present. Just before the COVID-19 pandemic (ie, 2017-2019), tracts with high sustained levels of structural racism past and present had, on average, over 2 more fatal overdoses per tract compared with sustained advantaged tracts (average marginal effect, 2.60; 95% CI, 2.02-3.19; *P* < .001). During the COVID-19 pandemic (2020-2022), tracts that were advantaged in the past but experienced high present-day segregation had, on average, almost 4 more fatal overdoses per tract compared with sustained advantaged tracts (average marginal effect, 3.81; 95% CI, 1.94-5.68; *P* < .001). The overall burden of overdose death was higher for all neighborhood groups during the pandemic compared with before the pandemic.

**Conclusions and Relevance:**

These findings provide preliminary evidence that structural racism could be a root cause of opioid-involved overdose deaths. Future research is needed to identify mechanisms linking structural racism to overdose deaths and to develop effective policies and programs to reduce fatal overdose rates.

## Key Points

**Question:**

To what extent is there a spatial association between neighborhood-level structural racism and opioid-involved overdose deaths?

**Findings:**

This cross-sectional study of 796 census tracts (2017-2019) and 792 census tracts (2020-2022) in Chicago, Illinois, found that neighborhoods that were exposed to high levels of structural racism in the past (historical redlining) and present (contemporary segregation) had the highest fatal overdose incidence rates before the COVID-19 pandemic (2017-2019). Neighborhoods that experienced high levels of contemporary racism had the highest fatal overdose incidence rates during the pandemic (2020-2022).

**Meaning:**

This serial cross-sectional study provides preliminary empirical evidence that heightened neighborhood-level structural racism could potentially be a driver of opioid-involved overdose deaths in urban settings.

## Teaser

This cross-sectional study uses a geospatial and intersectional analytic approach to investigate whether there is an association between neighborhood-level structural racism and opioid-involved overdose deaths among Black individuals in the US.

## Introduction

Racial and ethnic minoritized communities have been disproportionately affected by the US drug overdose epidemic during its third and fourth waves. The fatal drug overdose rate for non-Hispanic Black individuals increased by 44% from 2019 to 2020.^1^ Opioids have been the primary contributor to the increase in overdose deaths among non-Hispanic Black individuals.^2,3,4^ The US overdose crisis is partly due to a more dangerous illicit drug supply, including potent synthetic opioids such as fentanyl, disproportionately affecting racial and ethnic minoritized communities.^4,5,6^ Black men have been particularly affected by an increase in opioid-related deaths during the third and fourth waves of the US overdose epidemic.^7^

Scholarly efforts have underscored the urgent need to dissect the factors driving the disproportionate rates of fatal overdoses within racially minoritized communities. Structural inequalities, encompassing social, economic, and infrastructural dimensions, have been increasingly theorized as fundamental drivers of these disparities.^8,9,10,11^ A qualitative study by Banks and colleagues^12^ found that opioid overdose deaths among Black individuals in the US are often the result of unmet needs for safety, security, stability, and survival—needs that are unfulfilled due to structural disinvestment and systemic barriers rooted in structural racism. A national study of US counties found that having a higher amount of per capita revenue generated from fines and forfeitures—a novel measure of structural racism concentrated among low-income minoritized populations—was associated with higher county-level fatal overdose rates.^13^ Studies have reported racial inequities in access to medications for opioid use disorder (OUD) and naloxone. Neighborhoods that are less racially and economically segregated have greater access to buprenorphine compared with areas with high deprivation and racialized economic segregation.^14,15^ Moreover, non-Hispanic White individuals who use drugs are more likely to have access to buprenorphine, take-home methadone, and naloxone compared with Black and Latino individuals who use drugs.^15,16,17,18^ The “War on Drugs” and subsequent drug policies have disproportionately targeted racially minoritized communities, resulting in higher incarceration rates for Black people who use drugs compared with their counterparts who are not Black.^19^ Criminal justice involvement and incarceration are key determinants of overdose and are disproportionately experienced by Black people who use drugs.^20,21^ These carceral factors increase vulnerability to determinants of overdose, including housing instability and underutilization or poor retention in drug treatment.^22,23^ Studies find that incarcerated individuals are more likely to have unmet needs for OUD medications and less likely to use harm reduction strategies (eg, fentanyl test strips).^24,25^ The combination of different structural inequalities often leads to geographic clusters of fatal overdoses within disadvantaged, majority-Black neighborhoods.^26,27,28^ This pattern emphasizes the critical role of racialized environments in overdose mortality. It signals the necessity to better understand the complex sociospatial dynamics that may influence disproportionate opioid overdose deaths among racially minoritized communities. To date, few articles have examined indicators of structural racism and their association with substance use outcomes. Specifically, there is a gap in studies examining the intersection of racism, place, and fatal opioid overdoses. This study seeks to fill this gap by investigating the association between neighborhood-level structural racism and opioid-involved overdose deaths.

Structural racism operates within the neighborhood context both historically and contemporarily, affecting neighborhoods across a wide span of time through a series of explicit policies and subtler institutional biases.^29^ In the 1930s, a US government agency called the Home Owners’ Loan Corporation (HOLC) developed a discriminatory policy known as redlining. Redlining involved marking specific neighborhoods within cities as “high risk” for investment. These neighborhoods were predominantly inhabited by low-income and working-class people as well as Black and other racially minoritized individuals.^30^ This practice systematically deprived these areas of financial and other essential services, entrenching economic and social disadvantages.^31^ Racialized economic segregation is a contemporary form of structural racism that reflects an unequal distribution of socioeconomic status and resources across neighborhoods. This form of segregation significantly disadvantages racial and ethnic minority populations across various indicators, such as income, employment, education, housing, and health.^32^

This study adopts an intersectional and geospatial approach to investigate the association between neighborhood-level structural racism and opioid-involved overdose deaths in Chicago, Illinois. Chicago is an important place to examine the association between structural racism and overdose for several reasons. Chicago’s fatal overdose rate has steadily increased since 2010, reaching record-high levels in 2021.^33^ Moreover, in 2022, there were more opioid-related overdose deaths in Chicago than homicides and traffic crash fatalities combined.^33^ Sixty-five percent of those overdose fatalities were among non-Hispanic Black individuals.^33^ Although we know that Black individuals were disproportionately represented in this increase,^34,35,36^ less is known about the potential structural drivers of this increase, including neighborhood-level structural racism.^37^ Since the Great Migration, Chicago neighborhoods have been impacted by numerous social policies and processes that have contributed to stark disparities in social advantage across the city’s neighborhoods. Notably, some of these factors include historical redlining and racial violence, hypersegregation, racial covenants, and zero-tolerance policing.^38,39,40^ By taking an intersectional and geospatial approach in this study, we can study multiple policies and processes over time and place, as well as systems of oppression (eg, structural racism and structural economic inequality), to better capture the complex social, structural, and spatial dynamics potentially associated with overdose death.^41^

In this study, we integrate measures of historical redlining and contemporary racialized economic segregation to investigate how past and present neighborhood-level structural racism contributes to spatial disparities in opioid-involved overdose deaths in Chicago, Illinois. We examine this association during 2 time periods: before the COVID-19 pandemic (2017-2019) and during the pandemic (2020-2022). This intersectional analysis seeks to elucidate the extent to which neighborhood-level structural racism may have perpetuated overdose deaths in racially minoritized communities, particularly during a time of worldwide crisis. We hypothesize that the burden of fatal overdoses will be highest in neighborhoods that experience high past and present levels of structural racism.

## Methods

### Data Source

The Cook County Medical Examiner’s office data are publicly available and updated daily and include complete toxicology reports and Global Positioning System coordinates for where the overdose occurred.^42^ This study included only overdoses that occurred within the Chicago city limits and excluded overdoses occurring in the Cook County suburbs. We adopted this exclusion criterion because redlining—an indicator in the index—was a policy that predominantly impacted urban areas. Opioid-involved fatal overdoses were identified using text-based identification.^43^ We defined an opioid-involved overdose death as including an opioid of any type (eg, heroin, prescription opioid, and fentanyl) as the primary cause of death.^43^ Suicides and homicides were excluded from this analysis, as well as overdoses that did not involve any opioids, because environmental context and individual characteristics may differentially impact drug choice, route of administration, and associated overdose risk.^44,45,46^ The study did not require an institutional review board review as it did not include human participant research. We followed the Strengthening the Reporting of Observational Studies in Epidemiology (STROBE) reporting guideline for this research.

There is evidence that COVID-19 brought about a dramatic increase in overdose death rates across the US.^34^ To account for the potential varying impact of the COVID-19 pandemic on overdose deaths across Chicago neighborhoods, 2 separate analytic datasets were created for this study. The pre–COVID-19 dataset captures opioid-involved deaths before the COVID-19 pandemic, 2017 to 2019 (n = 2257). The peri–COVID-19 dataset captures opioid-involved deaths broadly during the COVID-19 pandemic, 2020 to 2022 (n = 3879).

### Measures

**Figure 1.**
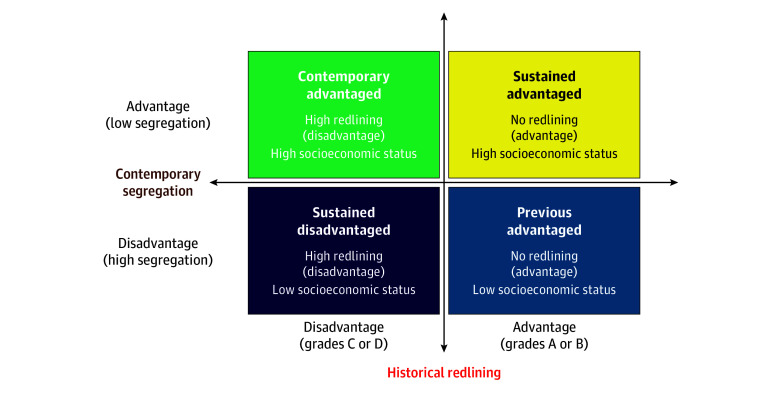
Description of Neighborhood-Level Trajectories of Structural Racism

We used a cross-classification method to develop an index that combined 2 forms of structural racism: historical redlining and contemporary racialized economic segregation. For further explanation of the cross-classification approach, see the study from 2023 by Uzzi et al.^47^ For historical redlining, we dichotomized the HOLC grades from the 1939 Chicago residential security map.^48^ We operationalized the redlining construct based on whether the census tract’s HOLC grade would be considered desirable (or not) for home loans and investment. HOLC assessors defined areas with high HOLC grades of “A” and “B” as the “best” and “still desirable” areas for investment, respectively. We classified these tracts as having no redlining. HOLC assessors defined areas with low HOLC grades of “C” and “D” as “definitely declining” and “hazardous” areas for investment, respectively; we classified these tracts as having high redlining. This study used the Historic Redlining Indicator dataset to assign a redlining grade for each census tract.^49^ For the pre–COVID-19 analysis, we used the Historic Redlining Indicator dataset for the 2010 US census tracts. For the peri–COVID-19 analysis, we used the Historic Redlining Indicator dataset for 2020 US census tracts. For contemporary racialized economic segregation, we generated dichotomous tract-level Index of Concentration at the Extremes (ICE) scores, using the median annual household income for non-Hispanic Black and non-Hispanic White households from the 2019 and 2022 5-year US Census Bureau’s American Community Survey for the pre–COVID-19 and peri–COVID-19 periods, respectively.^32^ This study’s ICE scores can be used as a proxy for determining whether the type of segregation within a census tract is advantageous or detrimental for the tract’s residents from the perspective of socioeconomic status and neighborhood resource allocation. The ICE measure quantifies the concentration of households in census tracts that are most socioeconomically advantaged (non-Hispanic White households with an income of ≥$100 000) vs those that are most socioeconomically disadvantaged (non-Hispanic Black households with incomes below the 2019 federal poverty line of $25 000). To classify sociospatial advantage and disadvantage with regard to contemporary racialized economic segregation, we used the median split of the ICE scores for the City of Chicago census tracts. Census tracts with an ICE score above the median split were defined as having high ICE scores (greater advantage), and tracts with scores below the median split were defined as having low ICE scores (greater disadvantage). We then created 4 intersectional groups of neighborhood-level structural racism in the study sample (Figure 1): (1) sustained advantaged (tracts that experience contemporary socioeconomic advantage and were not historically redlined); (2) sustained disadvantaged (tracts that experience contemporary socioeconomic disadvantage and were historically redlined); (3) contemporary advantaged (tracts that experience contemporary socioeconomic advantage and were historically redlined); and (4) previous advantaged (tracts that experience contemporary socioeconomic disadvantage and were not historically redlined). We had separate sets of neighborhood intersectional groups for the pre–COVID-19 and peri–COVID-19 periods due to changes in census tract boundaries during the 2020 decennial census.^50^ We excluded tracts not assigned a HOLC grade and/or had no households in the tract (eg, airport) (51 in the pre–COVID-19 period; 55 in the peri–COVID-19 period).

### Statistical Analysis

We conducted a quasi-Poisson spatial regression to assess the association between structural racism and opioid-involved overdose deaths at the neighborhood level. The spatial model accounted for residual spatial autocorrelation and population density. We conducted a spatial regression using an eigenvector spatial filtering method to control for residual spatial autocorrelation. We calculated population density as a rate—the number of residents within a tract per square mile. We did not adjust for other spatially oriented factors to prevent overadjustment bias and multicollinearity.^51^ We also used information from the spatial model to conduct average marginal effect (AME) calculations. These AME calculations were conducted to assist with the interpretability of the results. AMEs report the difference in the expected number of overdoses per census tract, on average, when comparing intersectional groups that experienced high levels of past and/or present racism (sustained disadvantaged, contemporary advantaged, and previous advantaged) with the most advantaged group (sustained advantaged). We accounted for residual spatial autocorrelation and population density in the AME calculations. Positive numbers for AME calculations signify that the intersectional groups that experienced high levels of past and/or present racism had a greater number of overdoses compared with the sustained advantaged group. This study used an α = .05, two-tailed significance threshold. For additional context of the study findings, we reported incidence rate ratio results from the spatial model (eTable in Supplement 1). We used R software, version 4.4.0 (R Project for Computing) to geocode and aggregate all opioid-involved overdose deaths to the census tract level. Data were analyzed from February 19, 2024, to July 3, 2025.

## Results

### Sociodemographic Descriptive Statistics for Neighborhood Intersectional Groups of Structural Racism

**Figure 2.**
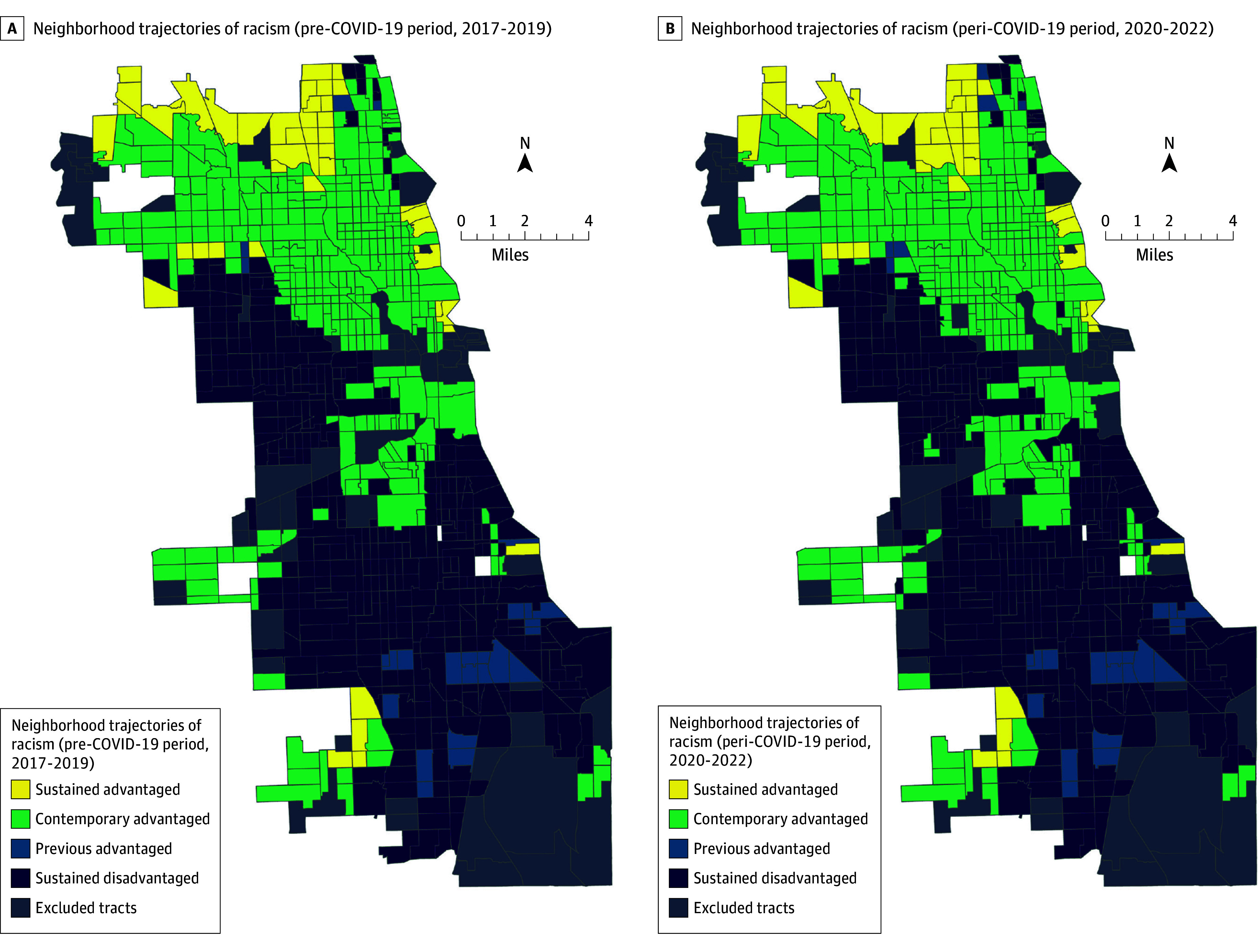
Map of Neighborhood-Level Trajectories of Structural Racism Before COVID-19 Pandemic (2017-2019) and During COVID-19 Pandemic (2020-2022) by Chicago, Illinos, Census Tracts

The total sample sizes for this serial cross-sectional study in Chicago were 796 census tracts for the pre–COVID-19 pandemic dataset (2017-2019) and 792 census tracts for the during COVID-19 pandemic (2020-2022) dataset. Among pre–COVID-19 census tracts, 44.8% (n = 357) were classified as sustained disadvantaged, 3.1% (n = 25) were previous advantaged, 39.9% (n = 318) were contemporary advantaged, 5.7% (n = 45) were sustained advantaged, and 6.4% (n = 51) of tracts were excluded from analysis (Figure 2). During the peri–COVID-19 period, 43.9% (n = 348) of census tracts were sustained disadvantaged, 3.4% (n = 27) were previous advantaged, 40.3% (n = 319) were contemporary advantaged, 5.4% (n = 43) were sustained advantaged, and 6.9% (n = 55) of tracts were excluded from analysis (Figure 2). During both the pre–COVID-19 and peri–COVID-19 periods, Black residents predominantly lived in the sustained disadvantaged areas and previous advantaged areas, whereas White residents predominantly lived in the sustained advantaged and contemporary advantaged areas (Table 1). Latino residents were spread throughout sustained advantaged, sustained disadvantaged, and contemporary advantaged areas. Moreover, there were higher average median household incomes in the sustained advantaged and contemporary advantaged areas compared with the previous advantaged and sustained disadvantaged areas during the pre–COVID-19 and peri–COVID-19 periods. The average median household income increased for all groups after the onset of the COVID-19 pandemic, ranging from a 22.3% increase in the sustained disadvantaged group to a 28.7% increase in the previous advantaged group.

**Table 1.** Descriptive Statistics of Chicago’s Neighborhood-Level Intersectional Group of Structural Racism, Before the COVID-19 Pandemic (2017-2019) and During the COVID-19 Pandemic (2020-2022)

Statistics	Before COVID-19 pandemic, 2017-2019					During COVID-19 pandemic, 2020-2022				
	Sustained advantaged (n = 45)^a^	Contemporary advantaged (n = 318)^a^	Previous advantaged (n = 25)^a^	Sustained disadvantaged (n = 357)^a^	Chicago average (n = 796)^b^	Sustained advantaged (n = 43)^a^	Contemporary advantaged (n = 319)^a^	Previous advantaged (n = 27)^a^	Sustained disadvantaged (n = 348)^a^	Chicago average (n = 792)^b^
Opioid overdose death rate per 10 000 residents^c^	0.74	1.45	3.07	4.65	2.76	1.22	2.30	6.79	8.17	4.72
Mean No. of fatal overdoses per tract (SD)	1.11 (1.21)	1.61 (1.79)	3.44 (2.31)	4.19 (5.00)	2.89 (3.96)	1.88 (1.68)	2.48 (2.93)	7.56 (5.29)	7.43 (7.58)	4.90 (6.17)
Race and ethnicity, %^d^										
Asian	13	9	4	3	7	13	9	4	3	7
Non-Hispanic Black	7	6	81	56	29	6	6	76	54	28
Hispanic or Latino	20	29	8	32	29	20	29	10	33	29
Non-Hispanic White	57	53	5	7	33	57	52	7	7	33
Median household income, mean (SD), $^e^	82 476 (29 373)	84 103 (33 062)	36 416 (10 860)	36 947 (14 703)	60 476 (34 493)	98 979 (31 577)	103 807 (39 298)	46 878 (13 731)	45 182 (16 875)	74 863 (41 651)
Population density, residents per square mile^f^	14 032	17 103	13 241	11 860	12 464	14 096	16 716	13 949	11 956	12 387

^a^ Number of census tracts in the group.

^b^ Number of census tracts in Chicago, Illinois, for 2017-2019 and 2020-2022. Tracts excluded from analyses (eg, tracts that did not have Home Owners’ Loan Corporation grades assigned to them or did not have household populations) were included in Chicago citywide averages to provide citywide context of variables.

^c^ Mean annualized opioid-involved death rates per 10 000 residents. Overdose data from Cook County Medical Examiners Office (2017-2022). Resident population data from 5-year 2019 and 5-year 2022 US Census Bureau American Community Survey.

^d^ The race and ethnicity variable from 5-year 2019 (pre–COVID-19 period) and 5-year 2022 (peri–COVID-19 period) US Census Bureau American Community Surveys, respectively.

^e^ The median household income variable from 5-year 2019 (pre–COVID-19 period) and 5-year 2022 (peri–COVID-19 period) US Census Bureau American Community Surveys. The table’s value represents the mean average of the median household income per group; for the 2020-2022 dataset, results from 11 tracts were missing.

^f^ Number of residents in entire neighborhood group per square mile. Average number of residents per neighborhood group and square mile size of neighborhood group from 5-year 2019 (pre–COVID-19 period) and 5-year 2022 (peri–COVID-19 period) US Census Bureau American Community Surveys.

### Opioid-Involved Overdose Deaths

**Figure 3.**
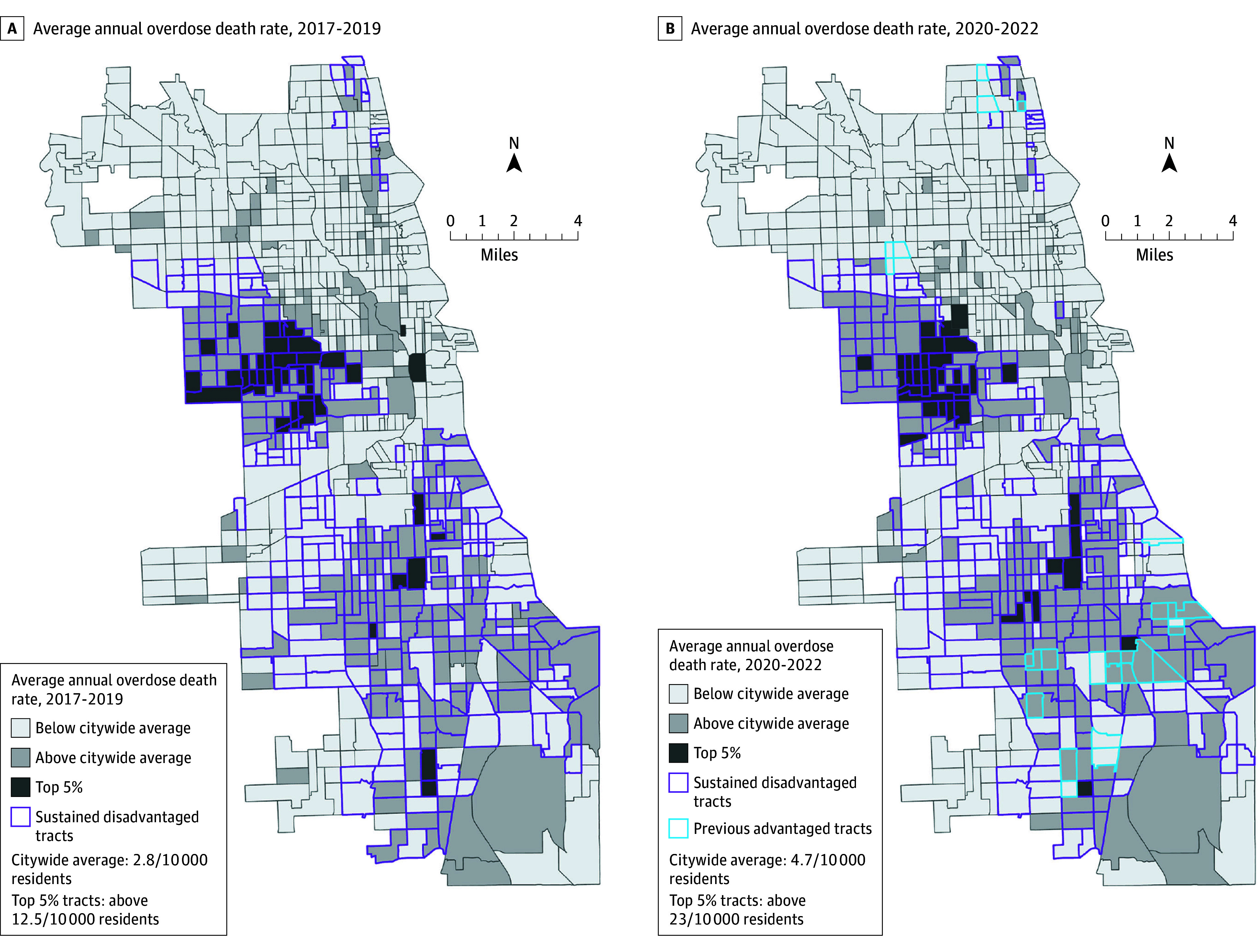
Maps of Average Annual Opioid-Involved Overdose Death Rates Before COVID-19 pandemic (2017-2019) and During COVID-19 Pandemic (2020-2022) by Chicago, Illinois, Census Tracts

The total number of opioid-involved death incidents analyzed in this study were 2257 before the COVID-19 pandemic (2017-2019) and 3879 during the COVID-19 pandemic (2020-2022). The overdose incidents are aggregated to the census tract level so that we can perform the spatial analysis. The mean rate for opioid-involved overdose deaths in Chicago was 2.76 per 10 000 residents per year during the pre–COVID-19 period and 4.72 per 10 000 residents per year during the peri–COVID-19 period (Table 1). The highest overdose death rates before and during the pandemic were in Chicago’s westside and southside (Figure 3). The mean overdose death rate pre–COVID-19 ranged from 4.65 per 10 000 residents for the sustained disadvantaged group to 3.07 for the previous advantaged, 1.45 for contemporary advantaged, and sustained advantaged 0.74 (Table 1). The mean overdose death rate during the peri–COVID-19 period ranged from 8.17 per 10 000 residents for the sustained disadvantaged group to 6.79 for previous advantaged, 2.30 for contemporary advantaged, and sustained advantaged 1.22 (Table 1).

During the pre–COVID-19 period, the sustained disadvantaged tracts, on average, had more expected overdoses per tract than the sustained advantaged tracts (AME, 2.60; 95% CI, 2.02-3.19; *P* < .001) (Table 2). The previous advantaged tracts had more overdoses (AME, 2.11; 95% CI, 1.01-3.20; *P* < .001) than the sustained advantaged tracts, and the contemporary advantaged tracts had more overdoses (AME, 0.95; 95% CI, 0.47-1.42; *P* < .001) than the sustained advantaged tracts.

**Table 2.** Average Marginal Effects of Opioid-Involved Overdose Death by Neighborhood-Level Intersectional Group of Structural Racism in Chicago, Before the COVID-19 Pandemic (2017-2019) and During the COVID-19 Pandemic (2020-2022)^a^

Time period	Average marginal effect (95% CI)	*P* value
Before COVID-19 pandemic, 2017-2019		
Intersectional group		
Sustained advantaged	[Reference]	
Contemporary advantaged	0.95 (0.47 to 1.42)	<.001
Previous advantaged	2.11 (1.01 to 3.20)	<.001
Sustained disadvantaged	2.60 (2.02 to 3.19)	<.001
During COVID-19 pandemic, 2020-2022		
Intersectional group		
Sustained advantaged	[Reference]	
Contemporary advantaged	0.62 (−0.48 to 1.72)	.27
Previous advantaged	3.81 (1.94 to 5.68)	<.001
Sustained disadvantaged	3.08 (1.94 to 4.21)	<.001

^a^ The average marginal effect represents the difference in the expected number of overdoses per census tract, on average, when comparing an intersectional group that experienced racism with the reference group (sustained advantaged) after holding population density and residual spatial autocorrelation constant.

During the peri–COVID-19 period, the previous advantaged tracts, on average, had more expected overdoses per tract than the sustained advantaged tracts (AME, 3.81; 95% CI, 1.94-5.68; *P* < .001), and the sustained disadvantaged tracts had more overdoses per tract than the sustained advantaged tracts (AME, 3.08; 95% CI, 1.94-4.21; *P* < .001) (Table 2). The contemporary advantaged tracts did not differ significantly from the sustained advantaged tracts (AME, 0.62; 95% CI, −0.48 to 1.72; *P* = .27).

## Discussion

This research presents a geospatial approach to investigating the underresearched area of structural racism and its association with opioid-involved fatal overdoses. This study used a combination of both historical and contemporary manifestations of structural racism to contextualize and understand how these intertwined dimensions of structural racism are associated with fatal overdose disparities in minoritized communities. We found significant disparities in overdose deaths in neighborhoods that experienced high levels of racism in the past and/or present (ie, sustained disadvantaged, previous advantaged, and contemporary advantaged) compared with sustained advantaged neighborhoods, which experienced low levels of racism between the 1930s and 2020s. Before the COVID-19 pandemic (2017-2019), neighborhoods that experienced present-day racism (sustained disadvantaged and previous advantaged tracts) had, on average, over 2 more fatal overdoses per tract compared with sustained advantaged tracts. Moreover, contemporary advantaged neighborhoods—characterized by high levels of historical redlining and lower levels of contemporary racialized economic segregation—had, on average, around 1 more fatal overdose per tract compared with sustained advantaged tracts.

During the pandemic period (2020-2022), we saw an increase in overdose death rates for all intersectional groups of neighborhood-level structural racism. This phenomenon of an increase in overdose deaths during the pandemic is consistent with previous research on the impact of the COVID-19 pandemic on overdose deaths, particularly among racially minoritized people and communities.^34,52^ We found that neighborhoods that experienced high levels of present-day structural racism (previous advantaged tracts and sustained disadvantaged tracts) had the highest overdose rates of all neighborhood groups during the COVID-19 pandemic. Moreover, the AME for previous advantaged and sustained advantaged tracts during the COVID-19 pandemic was over 3 fatal overdoses per census tract. This finding means that areas that experienced high levels of present-day structural racism had, on average, over 3 more overdose deaths per census tract compared with the sustained advantaged areas—when holding population density and residual spatial autocorrelation constant.

Residents of highly segregated neighborhoods, where the majority of residents are racially and economically marginalized, often face social and economic barriers. such as a lack of quality health care, education, employment opportunities, and reduced access to treatment services.^29,53,54,55,56^ A lack of access to these necessary social structures can lead to increased stress, exacerbate substance use, and increase vulnerability to determinants of overdose among residents of these neighborhoods.^56,57^ Racially minoritized people who use drugs can be particularly vulnerable to a combination of individual-level and historically rooted stressors—including structural racism—during times of crisis, such as the COVID-19 pandemic.^37,58,59,60^ The drug supply can also be hazardous in lower-income, urban, and Black communities, often being more lethal due to contamination with potent substances such as fentanyl.^6,26,36^ More research is needed to understand the types of drugs present in neighborhoods with higher levels of structural racism to provide a more nuanced understanding of opioid-related overdoses and the disproportionate impact of synthetic opioids and polysubstance use on specific neighborhoods and populations.^34,61^ This could include conducting retrospective research that compares experiences of nonfatal overdose based on neighborhood type and drug-use experience (eg, people with OUD vs other people who use drugs). This study provides preliminary empirical evidence that heightened neighborhood-level structural racism is associated with increased opioid overdose deaths, highlighting the necessity for further research to uncover the mechanisms connecting structural racism to overdose fatalities.

### Limitations

This study has limitations. This study investigated the association between neighborhood-level structural racism and fatal overdoses in 1 large US city. Future studies should use the intersectional approach to investigate this association in other cities to strengthen the external validity of the findings. Pandemic-related social distancing policies to prevent COVID-19 transmission may have influenced where people used opioids and overdosed. Future studies should include postpandemic overdose data to further investigate how structural racism impacts neighborhood overdose rates over time. Medical examiner records did not include home addresses; it is possible that individuals traveled from neighborhoods with low structural racism to high structural racism to purchase and consume drugs, further exacerbating neighborhood stressors and overdose disparities.^62,63^ Medical examiner records do not contain information on previous OUD diagnoses, limiting the ability to identify structural differences affecting fatal overdose among people with OUD vs people using fentanyl-adulterated drugs who do not have OUD.

## Conclusions

This cross-sectional study provides preliminary evidence that neighborhood-level structural racism could be a contributing root cause of opioid-involved overdose deaths. These findings suggest that there is an association of the intersection of historical policies (ie, redlining) and current social processes (eg, racialized economic segregation) with neighborhood-level fatal opioid overdose rates. Structural racism is an upstream problem that has long impacted neighborhoods in Chicago and throughout the US. Similar to previous research literature, we believe that policy changes that promote increased community-based structural investment in racially minoritized neighborhoods are essential to address systemic barriers that exacerbate overdose disparities.^12,13,64^ If properly implemented, these policies have the potential to dramatically improve the quality of and access to vital social and health services necessary for people who use drugs to engage in harm reduction strategies as well as foster healthier and supportive communities.^65,66,67^

The implementation of policies that improve access to health care has been shown to increase access to medication for OUD and naloxone distribution and decrease overdose deaths among vulnerable racialized minoritized groups.^68,69,70^ Moreover, implementing housing interventions could address both short-term and long-term overdose risks.^71,72^ Given the association between criminal justice involvement and overdose, more dedicated interventions are needed to address both the social and health needs for people who use drugs and who are involved in the criminal justice system across the criminal justice continuum.^73^ Furthermore, it will be crucial to mobilize a range of community, social, and health care stakeholders within neighborhoods that experience high levels of structural racism. These stakeholders can support individuals at increased risk for overdose by linking these individuals to social support services and health care interventions and challenging the structural stigma faced by racially minoritized people who use drugs.^74,75,76,77^ A range of local public agencies and nonprofit organizations have begun to mobilize and address Chicago’s overdose crisis through hyper-local, place-based approaches. The approaches include the Summer Opioid Response Incident Command Structure (SOR-ICS) and the Chicago Crisis Assistance Response and Engagement Overdose Response Team (CARE ORT).^33,78^ These coordinated efforts have seen promising results of a significant decrease in overdose rates within Chicago’s westside neigborhoods.^33^

More research is needed to examine the nuanced mechanistic pathways between the neighborhood environmental manifestation of structural racism and its impact on fatal opioid overdose. Moreover, further research is necessary to specify the factors associated with the varying burdens of overdoses among different neighborhood-level structural racism groups during times of major crises. By implementing targeted place-based interventions through a multisystemic public health approach, we can promote equitable access to solutions that address the social and structural determinants of health and reduce overdose fatalities among structurally vulnerable communities.

## Notes

Supplement 1. **eTable.** Incidence Rate Ratios of Opioid-Involved Overdose Death by Neighborhood-Level Intersectional Group of Structural Racism in Chicago; Before the COVID-19 Pandemic (2017-2019) and During the COVID-19 Pandemic (2020-2022)

Supplement 2.
Data Sharing Statement
