# Supplementary material for: Fatal Opioid Overdoses by Historical and Contemporary Neighborhood-Level Structural Racism
Source: JAMA Health Forum. 2025 Nov 7;6(11):e253986. doi: 10.1001/jamahealthforum.2025.3986 (PMC12595539; doi:10.1001/jamahealthforum.2025.3986)
Supplement: Supplement 1. — eTable. Incidence Rate Ratios of Opioid-Involved Overdose Death by Neighborhood-Level Intersectional Group of Structural Racism in Chicago; Before the COVID-19 Pandemic (2017-2019) and During the COVID-19 Pandemic (2020-2022) [file jamahealthforum-e253986-s001.pdf]

Supplemental Online Content

Uzzi M, Ricard JR, Belton I, et al. Fatal opioid overdoses by historical and contemporary neighborhood-level structural racism. *JAMA Health Forum*. 2025;6(11):e253986. doi:10.1001/jamahealthforum.2025.3986

eTable. Incidence Rate Ratios of Opioid-Involved Overdose Death by Neighborhood-Level Intersectional Group of Structural Racism in Chicago; Before the COVID-19 Pandemic (2017-2019) and During the COVID-19 Pandemic (2020-2022)

This supplemental material has been provided by the authors to give readers additional information about their work.

© 2025 Uzzi M et al. *JAMA Health Forum*.

**eTable.** Incidence Rate Ratios of Opioid-Involved Overdose Death by Neighborhood-Level Intersectional Group of Structural Racism in Chicago; Before the COVID-19 Pandemic (2017-2019) and During the COVID-19 Pandemic (2020-2022)*			
Before COVID-19 pandemic, 2017-2019			
Intersectional Group	Incidence Rate Ratio	95% Confidence Interval	p-value
Sustained Advantaged	Ref	Ref	Ref
Contemporary Advantaged	1.97	(1.31 to 3.10)	p= 0.002
Previous Advantaged	3.16	(1.86 to 5.48)	p< 0.001
Sustained Disadvantaged	3.67	(2.41 to 5.84)	p< 0.001
*Adjusted for population density and residual spatial autocorrelation			
During COVID-19 pandemic, 2020-2022			
Intersectional Group	Incidence Rate Ratio	95% Confidence Interval	p-value
Sustained Advantaged	Ref	Ref	Ref
Contemporary Advantaged	1.23	(0.84 to 1.88)	p= 0.31
Previous Advantaged	2.43	(1.56 to 3.87)	p< 0.001
Sustained Disadvantaged	2.15	(1.48 to 3.27)	p< 0.001
*Adjusted for population density and residual spatial autocorrelation			

© 2025 Uzzi M et al. *JAMA Health Forum*.
