# Supplementary material for: Fatal Opioid Overdoses by Historical and Contemporary Neighborhood-Level Structural Racism
Source: JAMA Health Forum. 2025 Nov 7;6(11):e253986. doi: 10.1001/jamahealthforum.2025.3986 (PMC12595539; doi:10.1001/jamahealthforum.2025.3986)
Supplement: Supplement 2. — Data Sharing Statement [file jamahealthforum-e253986-s002.pdf]

Data Sharing Statement

Uzzi. Fatal Opioid Overdoses by Historical and Contemporary Neighborhood-Level Structural Racism. *JAMA Health Forum*. Published November 07, 2025.
doi:10.1001/jamahealthforum.2025.3986

Data
Data available: No
